# Reliability of temperature signal in various climate indicators from northern Europe

**DOI:** 10.1371/journal.pone.0180042

**Published:** 2017-06-29

**Authors:** Pertti Hari, Tuomas Aakala, Emmi Hilasvuori, Risto Häkkinen, Atte Korhola, Mikko Korpela, Tapio Linkosalo, Harri Mäkinen, Eero Nikinmaa, Pekka Nöjd, Heikki Seppä, Mika Sulkava, Juhani Terhivuo, Heikki Tuomenvirta, Jan Weckström, Jaakko Hollmén

**Affiliations:** 1 University of Helsinki, Department of Forest Sciences, Helsinki, Finland; 2 Laboratory of Chronology, Finnish Museum of Natural History, University of Helsinki, Helsinki, Finland; 3 Natural Resources Institute Finland (Luke), Helsinki, Finland; 4 University of Helsinki, Department of Environmental Sciences, Helsinki, Finland; 5 Aalto University, Department of Information and Computer Science, Aalto, Espoo, Finland; 6 University of Helsinki, Department of Geosciences and Geography, Helsinki, Finland; 7 Natural Resources Institute Finland (Luke), Aalto, Espoo, Finland; 8 Natural Resources Institute Finland (Luke) Statistical services, Helsinki, Finland; 9 University of Helsinki, Finnish Museum of Natural History, Helsinki, Finland; 10 The Finnish Meteorological Institute, Helsinki, Finland; Universidade de Vigo, SPAIN

## Abstract

We collected relevant observational and measured annual-resolution time series dealing with climate in northern Europe, focusing in Finland. We analysed these series for the reliability of their temperature signal at annual and seasonal resolutions. Importantly, we analysed all of the indicators within the same statistical framework, which allows for their meaningful comparison. In this framework, we employed a cross-validation procedure designed to reduce the adverse effects of estimation bias that may inflate the reliability of various temperature indicators, especially when several indicators are used in a multiple regression model. In our data sets, timing of phenological observations and ice break-up were connected with spring, tree ring characteristics (width, density, carbon isotopic composition) with summer and ice formation with autumn temperatures. Baltic Sea ice extent and the duration of ice cover in different watercourses were good indicators of winter temperatures. Using combinations of various temperature indicator series resulted in reliable temperature signals for each of the four seasons, as well as a reliable annual temperature signal. The results hence demonstrated that we can obtain reliable temperature information over different seasons, using a careful selection of indicators, combining the results with regression analysis, and by determining the reliability of the obtained indicator.

## Introduction

The present global warming highlights the importance of detailed and reliable information on past climate to place the current change in a historical context, to predict the response of biological and physical phenomena to global warming [1], and to develop and assess the performance of climate models. Instrumental temperature measurements cover a relatively short period, usually less than 150 years. In addition, the observation network becomes sparser the further back in time we go, and the more remote the area of interest is. Thus, indirect temperature series are needed to expand the temporal and spatial coverage of temperature information.

Fortunately, there are now multiple indicators of climate apart from surface temperature measurements (e.g. [2, 3]). Some of these temperature indicators are used indirectly to obtain information on climate before the 19th century, the era of systematic temperature measurements. The indicators that usually comprise the longest data series of monitoring reflect the temperature during a certain period of time, such as a certain phase of the annual metabolic cycle in biological indicators or of the ice-cover cycle. However, the temperature information obtained via such indicators includes noise and bias [4], illustrating the need to evaluate the reliability of the temperature information of each indicator. Optimally, such assessment is done within the same statistical framework, to allow for an objective assessment of the reliability of their temperature signal.

Several natural phenomena and processes can be used as temperature indicators at high temporal resolution over varying time scales from biological archives spanning several millennia, to observational records spanning decades to several centuries. Such indicators include various tree ring properties, bud burst of leaves and flowering, and melting and freezing of seas, lakes and rivers. However, the characteristics and applicability of these various indicators differ.

Tree ring-width measurements are one of the most widely applied indicators of past temperatures. Their popularity has been both due to their wide spatial coverage, the relative ease of measurements, and their unrivalled temporal resolution. The strength of the relationship between tree ring-widths and temperatures varies spatially, but it also seems to change through time [5, 6]. There are numerous papers addressing the issues and difficulties related to the use of ring-widths in studying past climates (e.g., [4, 7, 8]), and this research has led to a discovery of a range of other features that can be extracted from tree rings. Similar to ring-widths many of the other features appear to contain temperature information and are datable to an annual resolution. For example, previous studies have shown that measurement series of latewood density or its inexpensive alternative the intensity of blue light reflection have higher correlation with the growing season temperature than ring-widths alone (e.g., [9–11]). In addition, carbon isotopes in tree-rings have been shown to correlate more strongly with summer temperature than either tree ring-widths or latewood density [12]. Although tree ring formation in the high latitudes occurs during a fairly short period of time in the summer [13], there are subtle differences in the period of formation of the various features, and in how preceding environmental conditions influence them. Hence, a combination of various tree-ring properties, such as carbon isotopes and latewood density, has been suggested as an improvement by providing a wider time window [14], as they are sensitive to different periods during the growing season.

Most plant species are dormant during winter at high latitudes. During the dormant period plant metabolism is rather inactive and the plants are not growing. The metabolism activates again in the spring, following increasing air temperatures and solar radiation. The initial growth of new cells occurs inside the buds, and thus bud development becomes visible only later as the buds burst, which is easy to observe. The timing of budburst reflects the temperatures before the event. Plant phenological events, especially those taking place in the spring (i.e. timing of flowering and leaf bud burst), are shown to be driven by ambient temperatures during a month or two preceding the event [15–20].

Ice dynamics, i.e. ice formation, break-up and duration and extent of ice cover data series also have high potential as a climatic indicator [21, 22]. In this case the data would allow inferences for autumn, winter and spring climates, which are highly warranted in studying past climates. Lake ice cover is the result of local weather conditions, especially air temperature [23–25], and numerous studies suggest the usefulness of lake ice phenology as an indicator of long-term climate variability and change [21,23,26–28]. The strengths of ice phenologies as a climate indicator include the broad spatial distribution of sites, the high resolution of the data, and the relative ease and precision of observing the freeze and breakup moments, and hence the duration of cover both directly and by satellite [21,29].

Some observational data on ice dynamics extend further back in time than instrumental temperature measurements, but there are also promising means to reconstruct ice conditions of lakes from the palaeolimnological records [28,30,31]. These sediment analyses can provide annual-to-millennial scale time series of changes in different biological communities composition, biogeochemical processes and in lake physical conditions suitable for analysing winter climate effects on lakes [32].

Different indicator series used to estimate the past climates typically reflect the temperature history of only a fraction of the whole year. Tree-ring features usually reflect the conditions during the growing period, while spring phenological events are driven by the temperatures of late winter and early spring. Lake and river ice duration apparently depends on the temperatures throughout the winter, but also late autumn and early spring temperatures influence ice dynamics [33]. Therefore, past temperature proxies based on different indicators are not straightforward to compare, and may show discrepancies during the overlapping periods. On the other hand, the temporal overlap and temporal difference in conditions that influence various indicators can also be used to produce more detailed picture of seasonal and annual temperature change as shown in various so-called multi-proxy reconstructions (e.g., [34,35]).

We have a versatile collection of these types of indicator time series available in Finland and the adjacent regions. In addition, we can make additional retrospective measurements, especially from tree rings and lake sediments. It is clear that some of these time series may reflect climatic features very closely, while some of them might be only loosely connected with temperature [36]. Assessing the reliability of the temperature signal in each such time series is vital for climate research, for identifying phenomena that might be sensitive to change, and for practical applications. Hence, we should know how much of the variance in the observed temperatures is explained by the temperature signal detected in the indicator series, during time periods when both are available. In several earlier studies from Fennoscandia, the correlations between temperature and various indicators have varied considerably, from 0.1 to 0.9 (e.g., [20,37–40]). If the reliabilities of the temperature signals in various indicators are not known then the indicators are often implicitly assumed to be equally good sources of information resulting in wrong conclusions in their applications. On the other hand, combinations of different indicators can be used for improvement in temperature signal, using multiple regressions (e.g., [41]). However, in those cases the problem is that the increase in the number of estimated parameters increases the risk of over-fitting [42], that is, the model describes the data set used in training the model, but does not generalize to a reliable predictive model. Considering the application of the temperature information contained in different indicators, the reliability of the regression models is paramount.

In this paper, we performed a detailed assessment of the reliability of temperature signal in various indicators, based on natural phenomena as climate indicators. Specifically, we (i) assembled all readily available and relevant biological and physical time series predominantly in Finland that are commonly considered potential climate indicators, along with relevant temperature measurements. We then (ii) identified the season of the climate that each series characterizes best, and (iii) determined numerically the reliability of temperature signal in each single indicator series, and in a combination of different series. With these analyses, we aimed to highlight the important aspect to obtain comparable results of reliability of various indicators and their combinations. We assessed all the time series within the same statistical framework to obtain comparable results.

## Material and methods

### Climate data

**Fig 1:**
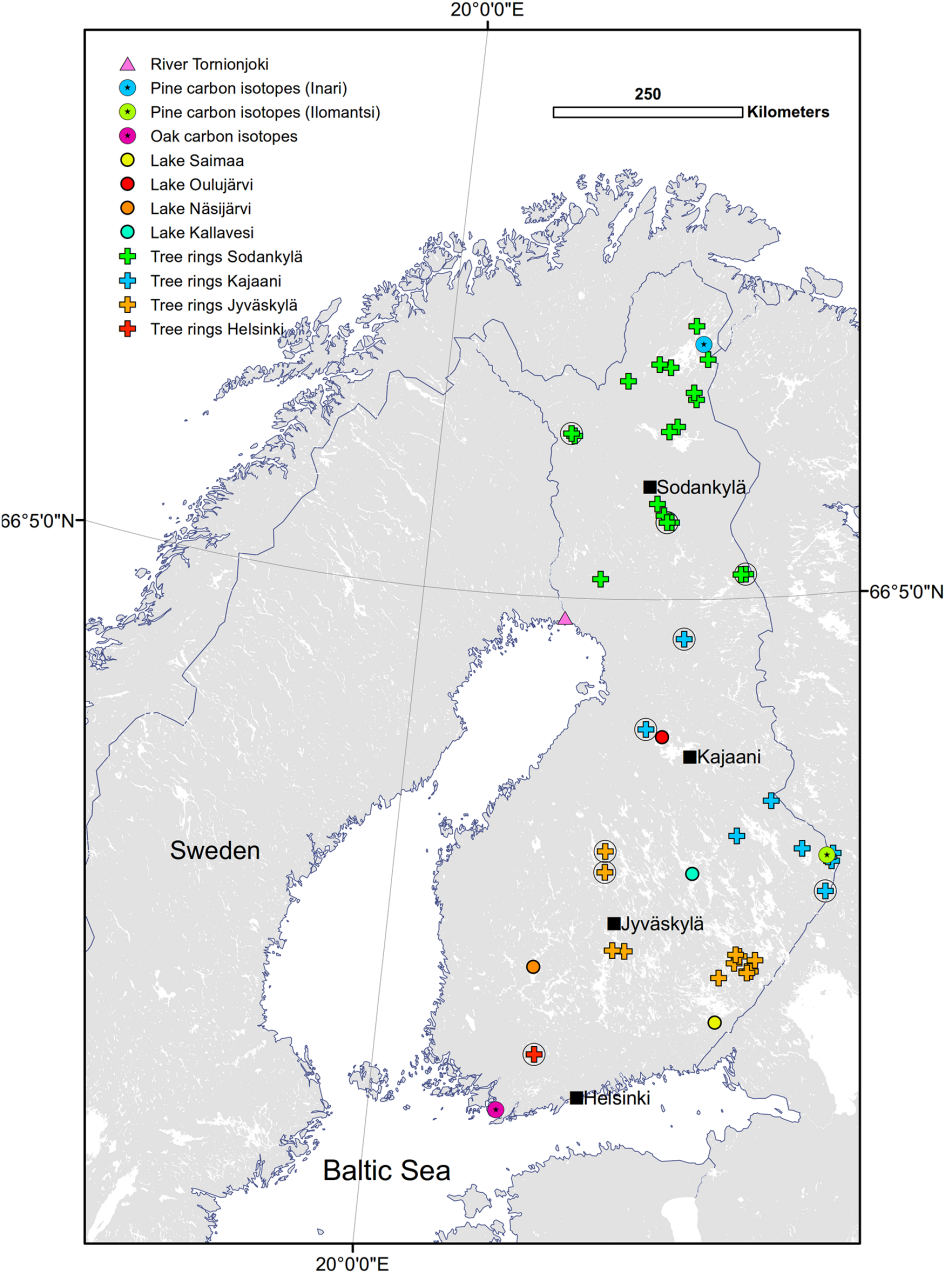
Map of the datasets used in this study. Location of meteorological stations (Helsinki, Jyväskylä, Kajaani, Sodankylä, black squares), lake and river ice phenology measurements, carbon isotope chronologies and the tree ring width data sets, grouped into regional chronologies according to the nearest meteorological station. Circled data sets denote latewood maximum density data for that particular location (in addition to ring-width data). Plant phenology data sets were regularly distributed throughout the country, and are hence not shown on the map.

To compute the relationship between the temperature records and potential temperature indicators, we first explored the spatial variability in temperature measurements from four meteorological stations located along a transect from southern to northern Finland (Helsinki, Jyväskylä, Kajaani, Sodankylä; Fig 1), all homogeneity tested and adjusted temperature time series. The time span covered by each of these series varied, but the measurements from all four stations between 1953 and 2004 were highly correlated (Helsinki and Jyväskylä 0.96, Kajaani and Jyväskylä 0.96, and Sodankylä and Jyväskylä 0.86). Hence, we utilized the longest continuous measurement series from Jyväskylä (since 1883; S1 Fig) in all further analyses. We used seasonal and annual mean temperatures as explanatory variables, when computing the relationships between the temperature records and the candidate temperature indicators.

### Tree rings

We used three types of tree-ring data sensitive to variations in temperature: tree ring-widths, latewood densities and the ratios of stable carbon isotopes (^13^C:^12^C) in the cellulose of tree rings. For tree ring-width data, we assembled all ring-width series from the International Tree Ring Data Bank (ITRDB; S1 Table) that originated in Finland and covered at least the time period 1850–1950. This way, there were at least 30 year-old trees from each data location (Fig 1) at the onset of the Jyväskylä climate records and a sufficient overlap with the records. These data included seven locations for Norway spruce (*Picea abies* (L.) Karst.) and 36 sites for Scots pine (*Pinus sylvestris* L.).

We compiled these data into several separate data sets. First, we divided the data to southern and northern Finland, the division being at 66.5°N latitude (Table 1). We applied this division because the northern forests are more open than the southern forests and hence tree growth in the north is less influenced by stand dynamics. It is usually assumed that the tree rings from the northern locations contain more temperature information. In addition, we created regional groups, by grouping the ring-width series based on their proximity to the weather stations. The regional groups may contain different amounts of temperature information potentially dampened in the geographically wider data.

**Table 1:** Temperature indicator series and weather data used in this study (see S2 Fig), along with sub-division of tree ring series to regional series (from South to North).

Object	Measurement / observation	Data type	First year	Last year	Data missing
Birch	bud burst	day of year	1846	2004	1876–1885, 1960, 1989
Bird cherry	flowering	day of year	1846	2004	1989
Rowan	flowering	day of year	1846	2004	1989
Lake Kallavesi	melting, freezing	day of year	1833	2009	
Lake Näsijärvi	melting, freezing	day of year	1836	2009	
Lake Oulujärvi (Vaala)	melting, freezing	day of year	1854	2009	
Lake Saimaa (Lauritsala)	melting, freezing	day of year	1885	2009	
River Tornionjoki	melting	day of year	1693	2009	
Baltic Sea	maximum ice cover	area	1720	2009	
Pine South	ring width	length	1573	2002	
Pine North	ring width	length	1410	2001	
Spruce South	ring width	length	1726	2001	
Spruce North	ring width	length	1701	1978	
Pine Helsinki	ring width	length	no datasets		
Pine Jyväskylä	ring width	length	1602	2002	
Pine Kajaani	ring width	length	1573	2001	
Pine Sodankylä	ring width	length	1410	2001	
Spruce Helsinki	ring width	length	1801	1978	
Spruce Jyväskylä	ring width	length	1815	2001	
Spruce Kajaani	ring width	length	1755	1978	
Spruce Sodankylä	ring width	length	1701	1978	
Pine South	latewood max. density	density	1643	1978	
Pine North	latewood max. density	density	1670	1978	
Spruce South	latewood max. density	density	1701	1978	
Spruce North	latewood max. density	density	1726	1978	
Pine Helsinki	latewood max. density	density	no datasets		
Pine Jyväskylä	latewood max. density	density	1643	1978	
Pine Kajaani	latewood max. density	density	1779	1978	
Pine Sodankylä	latewood max. density	density	1670	1978	
Spruce Helsinki	latewood max. density	density	1801	1978	
Spruce Jyväskylä	latewood max. density	density	1818	1978	
Spruce Kajaani	latewood max. density	density	1778	1978	
Spruce Sodankylä	latewood max. density	density	1701	1978	
Oak (Bromarv)	carbon isotope ratio	δ13C	1901	2002	
Pine (Ilomantsi)	carbon isotope ratio	δ13C	1658	2002	
Pine (Inari)	carbon isotope ratio	δ13C	1689	2002	
Helsinki	Temperature	temperature	1951	2004	some months in 1952
Jyväskylä	Temperature	temperature	1883	2009	march 1883
Kajaani	Temperature	temperature	1846	2009	1873–1886, several months in 1846
Sodankylä	temperature	temperature	1908	2009	

Following common dendroclimatological methods, we created chronologies of ring-width indices for each of these groups. First, we detrended the ring-width series, using a smoothing spline with a 50% frequency cut-off in 60 years [43], and calculated the ring-width indices by division. We then pre-whitened the indices, using residuals from autoregressive models (AR(1) in dplR [44]), and calculated mean chronologies as bi-weight means. We constructed separate chronologies for both species and all regions. We also tested the use of the Regional Curve Standardisation [45], but found only minor differences in the tree ring-width indices between the different standardization methods.

Similar to ring-width data, we obtained the latewood density data from the ITRDB; data were available for six Scots pine and four Norway spruce sites. We also divided these data types into two: southern and northern, the division being at 66.5°N latitude (Fig 1, Table 1), and created a chronology for both regions, using a similar procedure as for ring-widths.

For the carbon isotopes, we included two Scots pine chronologies, one from northern and one from central eastern Finland, and one oak (*Quercus robur* L.) chronology from southern Finland (Fig 1, Table 1, see [40] for details). In short, the chronologies were created by dating and hand-cutting the tree rings, and extracting α-cellulose from the wood [46]. The isotopic ratio was measured using an isotope ratio mass spectrometer in the Finnish Museum of Natural History, University of Helsinki. The change in isotopic composition of atmospheric CO_2_, which is caused by emission of anthropogenic carbon into the atmosphere, was corrected from the series [47]. The isotopic ratio is expressed as delta values (δ) in relation to the internationally accepted standard Vienna Pee Dee Belemnite.

### Phenological series

The Finnish Society of Sciences and Letters initiated the systematic collection of phenological observations in 1846 and the work was more recently continued in cooperation with the Finnish Museum of Natural History, University of Helsinki (see [48] for details). The data were collected primarily by laymen; therefore the geographical area of data collection is extensive, from the southern coast of Finland at 60°N to the Arctic Circle (66.3°N) and 20°E ‒ 31°E longitude. The data were collected, using annual phenomena of common natural and garden plant species. The number of observations varied for different species; the most abundant, such as leaf bud burst and flowering of common tree species, had over 7000 observations (Table 1). The observations were mostly collected under rural conditions and, therefore, the phenological data were not biased by the urban heat island effect.

Most of the longest phenological time series are of spring flowering and leaf unfolding events. These phenomena typically take place in the late spring or early summer. As the bud development takes several weeks or even months before the bud break occurs, and as the bud development is a phenomenon mostly driven by ambient air temperature [15,18–20], we expected the phenological time series to typically reflect the temperature conditions during the second quarter of the calendar year.

### Ice-cover data

The yearly ice-cover data for four lakes were derived from the Oiva database provided by the Finnish Environment Institute [49]. Lakes Saimaa, Näsijärvi and Kallavesi are located in southern Finland and Oulujärvi in northern central Finland (Fig 1). The melting and freezing days were based on visual inspection in which the melting day represents the day when the ice has disappeared from the observation site by the lake and the freezing day when a permanent ice cover had formed. The ice-cover duration is the time between the freezing day and melting day. We also utilized the ice breakup series from the River Tornionjoki, northern Finland [50].

The annual maximum ice extent of the Baltic Sea has been strongly correlated to the wintertime air temperatures over the region [51–54]. Those time series extend back to the year 1720 [55], although according to [56], most reliable observations begin only in the late 19th century.

### Determination of the reliability of temperature signal

**Fig 2:**
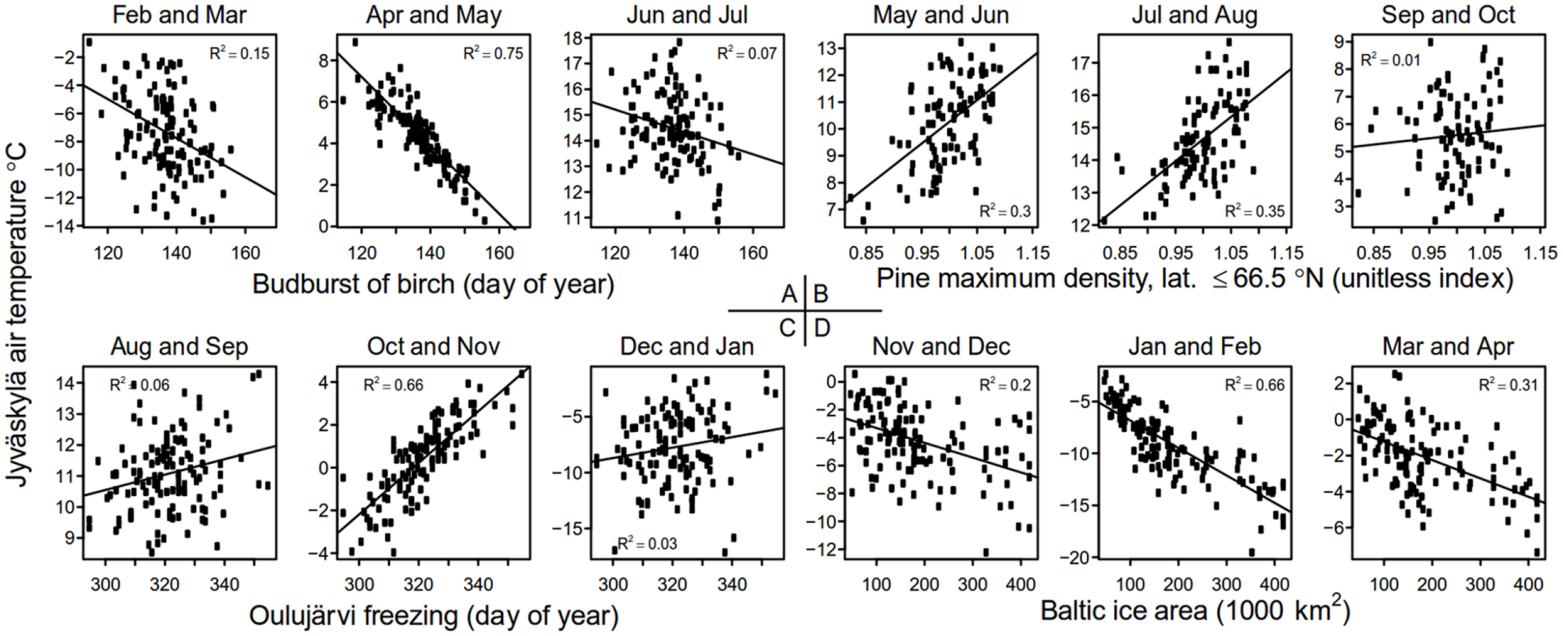
Scatter-plots between the mean temperature during two months at Jyväskylä and the timing of phenological and ice-cover events and tree-ring properties.

We determined the reliability of the temperature signal, using regression models between the indicator series and measured seasonal and annual temperatures from the meteorological station in Jyväskylä, since 1883. We detected no complex relationships between the observations or measurements and temperature in the preliminary analysis of the time series (Fig 2), and thus used linear regression models in the analyses [57]. We estimated the linear regressions between the seasonal mean temperatures and the individual indicators: timing of phenological events, freezing and melting of ice and duration of ice cover, and the properties of tree rings.

In addition to predicting temperature based on individual indicators, we also considered multivariate regression models, i.e., combining many indicator variables into a temperature predictor, using two approaches. First, as combinations of tree ring properties have been used in earlier studies (e.g., [41]), we tested the reliability of combinations of tree ring properties (ring width, maximum latewood density, carbon isotope composition). Second, to search for the most informative indicators for annual and seasonal temperatures across the indicator series, we used the least-angle regression (LARS; [58]), which has been used for quantifying the strength of the associations between environmental variables [59]. The method is similar to forward selection, but the values of the regression coefficients are adjusted more smoothly in LARS.

We estimated the reliability of the identified temperature signal based on the fit on a data set not used in the training. This mimics the situation in which unseen data is used to assess the performance of the model. We proceeded with the stepwise cross-validation approach in determining the proportion of the variance of the measured temperatures explained by the different indicators. In the cross-validation process, the original data were first split randomly into five non-consecutive parts, four of which were combined and used together as a training set and the remaining part as a validation set. This cross-validation procedure was repeated 50 times to eliminate the variation between the data sets used in the estimation. We considered 50 divisions sufficiently large for time series of this length to eliminate the effect of a single division to the results, while keeping the computation time reasonable.

The number of explanatory variables in the LARS models was selected using a second level of five-fold cross-validation within each training set of the first-level cross-validation described above, repeated 5 times. Each training set in the second level uses 80% of the corresponding first-level training set for training LARS models and the remaining 20% is used as a validation set for selecting the model complexity. We selected data that were not used for training when selecting model complexity, thus avoiding estimation bias, i.e., overfitting the model to the training data. For each training set in the first-level cross-validation, we selected the number of variables associated with the lowest sum of squared errors in the second-level validation sets.

Finally for comparison, full regression models including all the indicators were used to predict the temperatures. We averaged the prediction time series obtained using the first-level validation data and used the averaged time series to calculate the proportions of explained variance for these regression model types. We estimated the variation of the prediction time series using 10 000 bootstrap replicates.

The reliability of the indicator information was measured as one minus the ratio of the unexplained variance to the variance of temperature in the validation set. This approach is closely related to the coefficient of determination, i.e., the proportion of the variance explained, commonly used in regression analysis. The difference is that we only used the validation data set in the evaluation of reliability, avoiding estimation bias in the obtained reliabilities [60]. Neglecting separate validation data in the reliability analysis of temperature indicators may lead to overly positive or unfounded conclusions about the significance of the indicators [61,62]. Overly positive conclusions may also be caused by serial correlation, which decreases independence of a validation set in cross-validation [4], although this is not necessarily always the case in practice [63]. We found no serial correlations at lags of at least one year in the temperature data, or in most of the indicator series. However, the indicator series from Ilomantsi and Inari pine carbon isotopes showed serial correlation with a 1-year lag (0.51, and 0.42, respectively), and the River Tornionjoki ice phenology showed a slightly weaker serial correlation of 0.31. As discussed by Christiansen and Ljunqvist [4], this may cause additional uncertainty in the reliability measures of those indicators.

## Results

**Fig 3:**
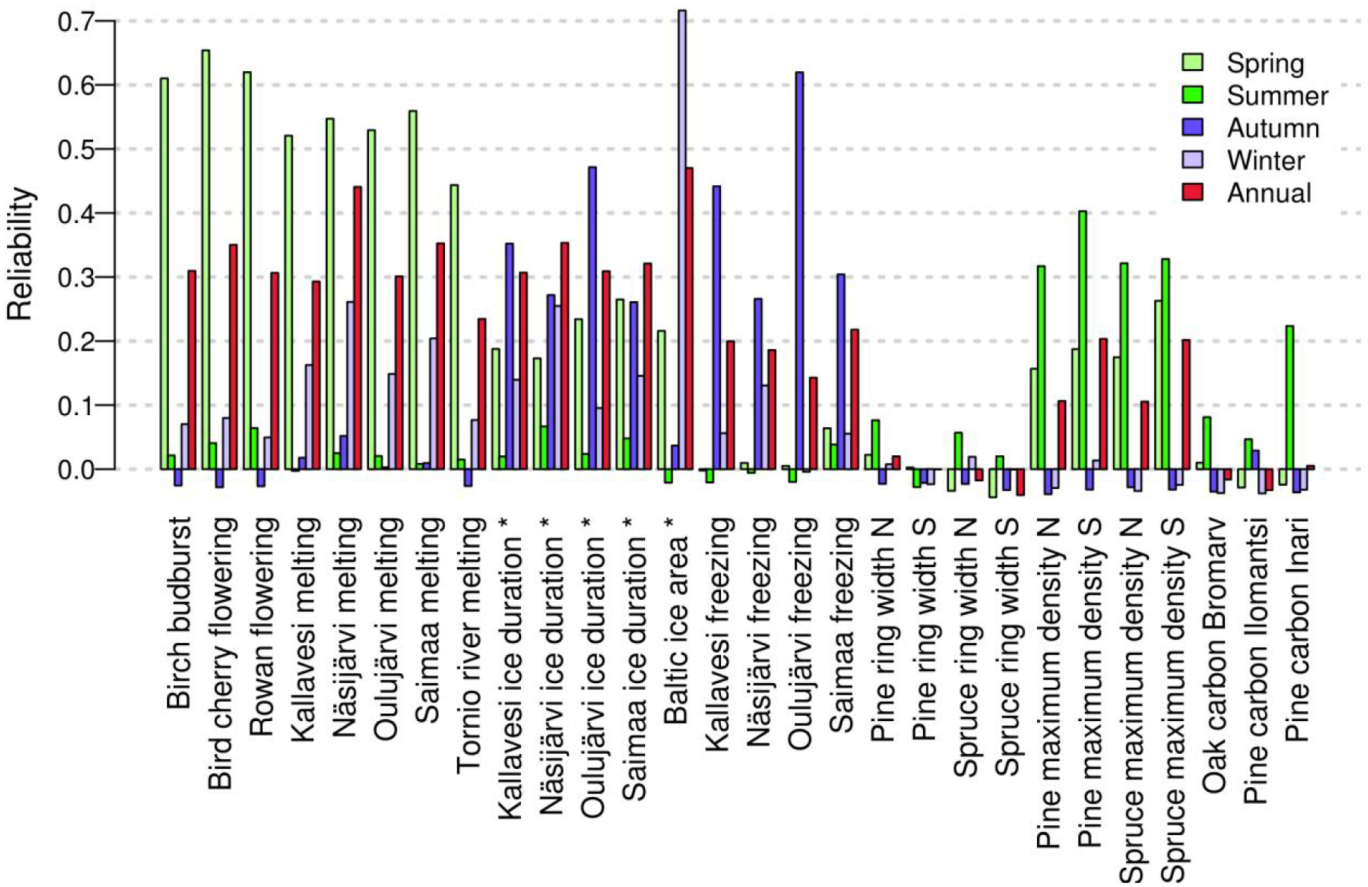
Reliability of individual indicators in predicting the seasonal temperature in Jyväskylä. Spring (March, April, May, light green), summer (June, July, August, green), autumn (September, October, November, blue), winter (December, January, February, light blue), and annual temperatures (red). Reliability is the proportion of variance explained by the averaged prediction time series in the validation set. The symbol * denotes usage of 2 consecutive years of time series data for annual prediction.

The reliability of temperature signal obtained from individual indicator time series varied within the year (Fig 2). Analyzed seasonally, spring (March, April, May) temperatures were strongly correlated with the plant phenological observations, the reliability being over 0.60 for birch bud burst, bird cherry flowering and rowan flowering (Fig 3). The melting dates of the lakes provided slightly less reliable information on spring temperatures (0.52–0.56), followed by the 0.44 of the River Tornionjoki melting. Ice cover duration of the lakes provided some information on spring, autumn (September, October, November) and winter (December, January, February) temperatures, with the reliability ranging between 0.20 and 0.30. The freezing dates of the lakes carried the strongest autumn temperature signal, especially for Lakes Oulujärvi and Kallavesi. However, the ice area of the Baltic Sea had the most reliable temperature signal among all indicators for predicting winter temperatures, with a reliability of 0.72.

The reliability of the temperature signal in indicators based on the ring-widths was low for summer (June, July, August) temperatures (reliability < 0.08) (Fig 2). Latewood density had a more reliable summer temperature signal (reliability: 0.32–0.40). Similar to the ring-widths, the temperature signal in the carbon isotopes from central Finland was unreliable. The carbon isotope chronology of Scots pine from northern Finland had somewhat more reliable temperature signal, with a reliability of 0.22.

**Fig 4:**
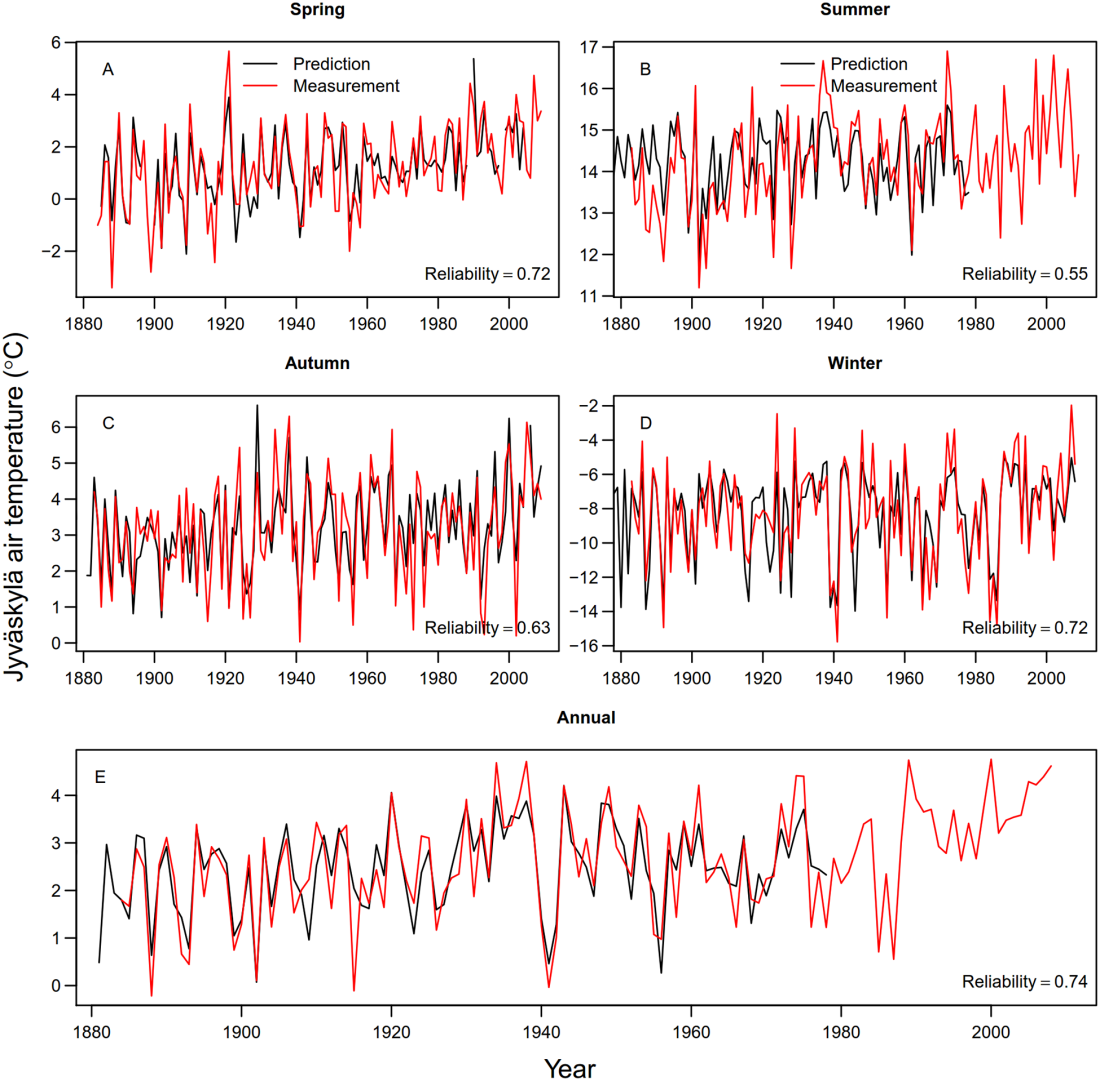
Time-series plot of predicted and measured seasonal (A-D) and annual (E) temperatures. Red line denotes the measured temperature, black line the predicted temperature, based on the best models: single variable for winter and LARS for spring, summer, autumn, and annual. All predictions were obtained using cross-validation. Missing predictions are due to lack of values in the indicator variables.

The combination of carbon isotopes, latewood density and ring-width data in the same model produced a reliable summer temperature signal, resulting in a reliability of 0.55 (Fig 4, Table 2). Similarly, even though the individual plant phenological and ice chronologies already carried reliable temperature signal on their own, combinations of several indicators further increased the reliability of temperature signal for spring and autumn temperatures (Fig 4, Table 2). Accordingly, we obtained reliable indicators for winter and, especially, for the whole year temperature by using a combination of different indicators. On the other hand, the results of the full models (Table 2) suggest that having too many indicators in a model decreases the reliability of the temperature signal.

**Table 2:** Reliability of temperature signal in the temperature indicator series, followed by 95% confidence intervals (in parentheses).

	LARS models				Full models				Best single				
Predicted output	R^2^		RMSE		R^2^		RMSE		R^2^		RMSE		Best indicator
Spring	**0.72**	(0.63–0.78)	0.83	(0.71–0.94)	0.47	(0.19–0.65)	1.09	(0.92–1.27)	0.65	(0.54–0.73)	0.93	(0.83–1.04)	Bird cherry
Summer	**0.55**	(0.38–0.66)	0.75	(0.66–0.84)	0.14	(-0.32–0.42)	1.05	(0.91–1.19)	0.41	(0.22–0.54)	0.87	(0.76–0.97)	Pine ring maximum density, Kajaani
Autumn	**0.63**	(0.53–0.71)	0.83	(0.72–0.94)	0.38	(0.05–0.60)	1.14	(0.95–1.33)	0.62	(0.52–0.70)	0.84	(0.73–0.95)	Lake Oulujärvi freezing
Winter	0.67	(0.59–0.73)	1.62	(1.43–1.81)	0.23	(-0.15–0.46)	2.35	(2.02–2.67)	**0.72**	(0.61–0.79)	1.51	(1.32–1.70)	Baltic Sea max ice area
Annual	**0.74**	(0.63–0.82)	0.56	(0.46–0.66)	0.53	(0.29–0.69)	0.74	(0.61–0.87)	0.47	(0.32–0.58)	0.81	(0.72–0.91)	Baltic Sea max ice area (two years)

In the table, LARS refers to the sparse models identified with the LARS algorithm. LARS selected the variables from the set of indicator variables that were all used in the full model. Best single is the best-performing variable in the temperature reconstruction. The best model for each season in terms of R^2^ of validation data is in boldface. Spring means March, April and May; summer June, July and August; autumn September, October and November; winter December, January and February. The confidence intervals are based on 10 000 bootstrap replicates of the analysis.

Based on the LARS models, the most informative combination of explanatory variables for spring (March, April, May) temperatures (Fig 4) were the melting dates of lakes Näsijärvi and Saimaa, bird cherry flowering and rowan flowering, and latewood maximum density of Scots pine in the Sodankylä region, each variable occurring in over 90% of the individual LARS models. The reliability of this combination of variables was 0.72. For summer temperatures, the most frequent variables (over 90%) were carbon isotopes in tree rings from all three sites, latewood maximum density of Scots pine in the Jyväskylä and Kajaani regions and ring-widths of Norway spruce in the Kajaani region. The reliability of this combination was 0.55. The reliability of the most informative combination of indicators (each in over 90% of the individual LARS models) predicting autumn temperatures were the freezing dates of Lake Oulujärvi and the carbon isotopes of oak tree-rings in southern Finland (reliability = 0.63). The next two informative variables for autumn temperatures (each in over 85% of the models) were the maximum latewood density of Norway spruce in Sodankylä and Baltic Sea ice extent, making a total of four variables (reliability = 0.68). For winter temperatures, the most frequent variables were the freezing dates of Lake Näsijärvi and the ice area of the Baltic Sea. These two variables occurred in 100% of the individual LARS models, and the reliability of this prediction was 0.67. The most informative combination of indicators predicting annual temperatures were the freezing dates of lakes Näsijärvi, Oulujärvi and Kallavesi, Baltic Sea ice extent, birch cherry flowering, ring-widths of Norway spruce in northern Finland, the melting dates of lake Näsijärvi, carbon isotopes in tree rings in northern Finland and maximum latewood density of Norway spruce in southern Finland. The reliability of this combination was 0.74.

## Discussion

In this study, we analyzed the reliability of temperature signal in various commonly used temperature indicators. These indicators differ in their characteristics, including their sensitivity to climate, measurability and feasibility, and spatiotemporal scope (cf. [1]). Hence, their applicability to obtain information on changing temperatures in the past and to reconstruct past temperatures varies.

Regardless of the application, an important aspect of these type of indicator data is their reliability. The use of a common statistical analysis framework in all our time series ensured comparability between the reliabilities of the different indicators. The use of separate validation data was a characteristic feature of the method we used for assessing the reliability of the temperature signal in the various indicators. This cross-validation procedure ensured that estimation bias did not increase artificially the reliabilities, and the obtained values contained useful, generalizable information on their reliability. In addition, the reliability allows not only comparing reliabilities of the temperature signal in single indicators but also reliabilities of multiple indicators without estimation bias. As a minor caveat, it should be noted that the serial correlation that was present in three of our indicator series (pine carbon isotopes in Inari and Ilomantsi, and to a lesser extent the River Tornionjoki ice phenology) may have led to slightly overestimated reliabilities [4, 63].

The temperature indicators analyzed carried information on the temperature conditions during and before their occurrence or formation. Different indicators capture the temperature signal from different parts of the year: ice melting moments and phenological events were related to spring temperatures, tree ring properties to summer temperatures, the freezing of lakes to autumn temperatures and the duration and extent of ice cover to winter temperatures.

For most of the indicators, especially those that are purely physical phenomena, the mechanisms connecting temperature and the indicator are well understood. As regards the ice dynamics, the energy pool explains the timing of melting and freezing moments [64–66]. The melting of ice on water bodies requires 333 kJ kg^-1^, and this energy is obtained from solar radiation and warm air. As an example, assume an ice covered lake with a 0.5 m thick ice cover, requiring over 16 000 kJ m^-2^ to melt. This energy is obtained during ~10 cloudless days if all energy comes from solar radiation. In reality, the snow cover and ice albedo are of importance to the energy balance. The cloudiness and temperature in spring varies greatly between years, and this results in variation in the availability of energy for melting and hence to variation in the dates of ice melting.

Compared to the melting of ice, the freezing of water bodies is a simpler phenomenon since the solar radiation plays a minor role in the energy budget of the surface water in late autumn. The cooling of surface water and finally the freezing is caused by the heat flux from the lake into cold air. In addition, evaporative cooling, and stream outflow are significant processes [67]. Freezing of lakes (especially Oulujärvi) carried thus reliable information on autumn temperatures.

Many similar processes that govern freezing and melting dates govern the Baltic Sea ice extent and the ice duration in lakes. This is reflected in the correlations between these indicators and autumn and spring temperatures. However, these indicators respond to temperatures differently as the Baltic Sea ice extent was the most reliable indicator for winter temperatures (reliability = 0.72) and ice duration in lakes predicted most reliably the autumn temperatures.

Although less straightforward compared to ice dynamics, the connection between timing of phenological events (flowering and bud burst) and temperatures is well understood. Our results were consistent with earlier studies that have reported temperature as the most important environmental factor controlling the timing of bud burst (e.g., [19,20]). These events require synthesis of new macromolecules and cell division. Increasing temperatures in spring accelerate this synthesis, giving rise to the connection between timing of phenological events and the antecedent temperature conditions. This straightforward connection was well visible in the reliability of these observations as spring temperature indicators. The bud burst and flowering of trees carried the most reliable spring temperature signal, reliability being between 0.61 and 0.65.

Tree rings are perhaps the most widely used source of temperature indicators, especially in reconstructing past temperatures. Temperature affects all metabolic processes involved in the formation of tree rings, and thus several properties of tree rings reflect the temperature conditions during and preceding the formation of tree rings. The formation of a tree ring is a complex process as it includes several connected phenomena, such as synthesis of macromolecules, cell division and maturation of cells. Cell enlargement is also directly depending on tree water status [68], further complicating the connection with temperature conditions.

Of various tree ring characteristics, ring-widths are the most commonly used indicators. In northern Europe, they have been used for past summer temperature reconstructions spanning centuries or millennia [69–71]. However, similar to our results here, numerous studies have demonstrated that the temperature signal contained in ring-widths alone can be fairly unreliable (reliability here between -0.03 and 0.08; e.g. [72]) and at least in some regions temporally unstable [5]. On the other hand, this is expected as ring-width integrates the tree’s responses to environmental conditions over longer time intervals due to, for instance, the trees’ capacity to store carbohydrates [13,73], the longevity of the photosynthetic machinery in coniferous trees [74] and changes in photosynthate allocation patterns with changing environmental conditions (e.g., higher allocation to roots during droughts; [75]).

Similar to previous studies (e.g., [71,76]), latewood density contained a stronger summer temperature signal than the ring-widths (reliability = 0.32–0.40). As maximum latewood density is related to both the tracheid dimensions and the cell wall thickness, it combines temperature information during the consecutive periods of tracheid enlargement and thickening of secondary cell wall (e.g., [13]).

A third tree ring characteristic used as a temperature indicator was the carbon isotope composition. The temperature signal in the carbon isotopes of oak and Scots pine tree-rings from southern and central Finland (Bromarv and Ilomantsi) was weak (reliability = 0.05–0.08) compared to the maximum latewood density. The carbon isotope chronology from northern Finland was more sensitive to temperature variation than the southern chronologies (reliability = 0.22), but was still a poorer temperature indicator than latewood density. Similar to some other tree ring-based indicators [6], McCarroll et al. [12] noted that the temperature signal showed differences depending on the time period analyzed. In their analyses carbon isotope composition was a poor temperature indicator prior to year 1917, consistent with our results here. However, McCarroll et al. [12] found that from year 1917 onwards, the carbon isotope composition was a good predictor for summer temperatures. For comparison, we repeated our analysis separately for the carbon isotope data from 1917 onwards. The reliability of Scots pine carbon isotopes from northern Finland and the oak carbon isotopes from southern Finland was lower after 1917 than in the entire time series (northern pines 0.22 vs. 0.18, southern oaks 0.08 vs 0.01), thus in contrast with the findings of McCarroll et all [12]. There was a clear improvement in the reliability of the pine carbon isotope chronology from eastern central Finland (from 0.05 to 0.20), but the reliability was still lower than for the latewood density. In addition, the carbon isotope indicators for pines in Inari and Ilomantsi contained serial correlations, which may have led to overestimates of their reliability [4]. The processes that lead to differences in carbon isotope ratios among years are not yet fully understood, but some studies indicate that carbon isotope chronologies register changes in solar radiation rather than temperature [77–79]. Solar radiation records in southern Fennoscandia may not be as strongly coupled with temperature as in northern Fennoscandia. However, we stress that the carbon isotope data used here came from a relatively small number of trees. A more reliable evaluation would require greater replication and sampling of trees from several sites.

Using a versatile collection of indicator series provided temperature information for various periods during the year, but their combination proved a reliable indicator of temperatures also at the annual level (Fig 4E). Despite the low reliability of individual characteristics in tree rings, combining the three characteristics (ring width, maximum density, carbon isotopic composition) improved the reliability as summer temperature indicator (reliability = 0.57). Several studies (e.g., [41,80]) have used multiple regression to combine the tree ring characteristics to a temperature indicator, and the proportion of explained variance is given for indicating the goodness of the fit. In this kind of cases care should be taken in the interpretation of the results, as the increasing number of indicators may improve the model fit, but may lead to the reliability of the model being biased, especially if the number of estimated parameters is too high.

However, it is noteworthy that low reliability of a given indicator does not mean that it is useless for climate change research. Even indicators with low strength of temperature signal may turn out useful, if the signal they contain is not present in other indicators, thus increasing the total reliability of obtained temperature signal. Proper application of these temperature indicators requires information on the season it indicates and on the reliability of the temperature signal in each indicator or in each indicator combination.

The physical and biological phenomena behind the formation of each of these temperature indicators is not specific to the region where our data originates from, so the finding that a selected and tested combination of indicators perform well in predicting the temperature of different seasons is applicable to other regions. As an example, the usefulness of plant phenological time series that performed well as a temperature indicator here, has been used elsewhere in Europe to demonstrate that in Europe leaf unfolding has advanced six days since the early 1960s due to spring warming [19,81]. However, especially considering reconstructions of past temperatures, the availability of documentary records (such plant and ice phenology) that were good indicators for spring, autumn, winter and annual temperatures is in many cases rather limited (however, see [3] for a number of potential understudied sources of documentary information). Nonetheless, as a climate change indicator or a monitoring target, phenological changes in trees or the timing of ice phenology have been considered one of the most sensitive to change [82], and they hold high potential as observations are quick and easy to make, and the spatial coverage of indicators in this category is broad.

Even if documentary records offer somewhat limited opportunities for past reconstruction of temperature, indicators from biological archives hold plenty of further potential [3]. Findings that other, less studied features (e.g., tracheid radial diameter, microfibril angle) in tree rings contain a reliable temperature signal offer potential for further developing tree-ring-based temperature indicators, as well as their spatial coverage [83,84]. In addition, development of standardization methods for removing variations unrelated to climate and better retaining low-frequency variation (e.g., [85,86], is improving the reliability of temperature reconstructions over extended periods of time (e.g., [87]).

A second promising source of future potential in biological archives is related to the ice phenology series that we found to be contain a temperature signal for autumn, winter, spring and annual temperatures. Longer ice phenology series would be a valuable addition to the commonly used biological indicators, which usually stress the active period during spring and summer. This is especially important because the winters are expected to exhibit the most prominent temperature increase [88]. Excluding the River Tornionjoki ice melting series since 1693, most of these time series unfortunately start about the same time as the instrumental temperature measurements. However, the length of ice cover can drive changes in key limnological variables including nutrient levels, mixing regimes, gas exchange, and, in poorly buffered sites, fluctuations in lakewater pH that are able to be inferred from biotic and abiotic proxies [89]. Accordingly, a number of recent studies have demonstrated the potential in extending past temperature reconstructions by means of paleolimnological techniques (e.g., [28,31,90,91]). When these novel findings are connected with careful analysis of the reliability of the obtained reconstruction, they offer further potential for reconstructing past climate, especially temperatures of other seasons besides summer.

## Conclusions

We studied the reliability of temperature signals in a collection of biological and physical indicators within the same statistical framework, using cross-validation methodology. This approach diminishes the adverse effect that estimation bias can have on the generalizability of our findings, as the cross-validation is not prone to overfitting and results in estimates of the true reliability of the indicators.

Our analysis revealed the reliabilities of temperature signals in different indicators for each of the four seasons, as well as for the annual temperatures. Plant phenologies and ice break-up dates were reliable indicators of spring temperatures and tree ring properties carried a reliable summer temperature signal when several of these properties were combined. Lake freezing dates carried reliable autumn temperature signal and ice area of the Baltic Sea performed well as a winter temperature indicator. Using a combination of various indicators increased temperature signal reliability.

We are facing the problem of missing temperature measurements before the construction of systematic network of temperature measurement stations. The temperature indicators are urgently needed to obtain climatic information before the systematic measurements since then we can properly assess the present warming, its strength and rate in the historical perspective. The weak knowledge of the climate in the past has undermined the efforts in placing the recent temperature changes into historical context. The unknown reliability of the temperature signal in the used indicators has been the dominant reason for the confusion in the discussion. The reliability should determine the weights of the indicators series in the argumentation.

## Supporting information

S1 Table Tree ring data sets used in this study. All data sets were obtained from the International Tree-Ring Data Bank (ITRDB). The search query covered all data sets that originated in Finland, and cover the time period 1850–1950. The series are listed from south to north.
(DOCX)

S1 Fig Jyväskylä monthly temperatures. (PDF)

S2 Fig Time series plots of individual indicators. (PDF)
