# Supplementary material for: Reliability of temperature signal in various climate indicators from northern Europe
Source: PLoS One. 2017 Jun 29;12(6):e0180042. doi: 10.1371/journal.pone.0180042 (PMC5491121; doi:10.1371/journal.pone.0180042)
Supplement: S1 Table — All data sets were obtained from the International Tree-Ring Data Bank (ITRDB). The search query covered all data sets that originated in Finland, and cover the time period 1850–1950. The series are listed from south to north. (DOCX) [file pone.0180042.s001.docx]

**S1 Table.** Tree ring data sets used in this study. All data sets were obtained from the International Tree-Ring Data Bank (ITRDB). The search query covered all data sets that originated in Finland, and cover the time period 1850-1950. The series are listed from south to north.

| **Lat (°N)** | **Lon (°E)** | **Species** | **First year** | **Last year** | **MXD** | **Data set id (in ITRDB)** | **Authors** |
| --- | --- | --- | --- | --- | --- | --- | --- |
| 60.67 | 23.88 | *P. abies* | 1801 | 1978 | yes | noaa-tree-4490 | Schweingruber, F.H. |
| 61.6 | 28.38 | *P. sylvestris* | 1850 | 1993 | no | noaa-tree-4012 | Meriläinen, J., Lindholm, M., Timonen, M., Huttunen, P. |
| 61.65 | 29.08 | *P. sylvestris* | 1786 | 1993 | no | noaa-tree-3982 | Meriläinen, J., Lindholm, M., Timonen, M., Huttunen, P. |
| 61.67 | 29.08 | *P. sylvestris* | 1774 | 1993 | no | noaa-tree-4007 | Meriläinen, J., Lindholm, M., Timonen, M., Huttunen, P. |
| 61.67 | 29.17 | *P. sylvestris* | 1783 | 1993 | no | noaa-tree-4015 | Meriläinen, J., Lindholm, M., Timonen, M., Huttunen, P. |
| 61.77 | 28.77 | *P. sylvestris* | 1704 | 1993 | no | noaa-tree-3985 | Meriläinen, J., Lindholm, M., Timonen, M., Huttunen, P. |
| 61.8 | 29.3 | *P. sylvestris* | 1799 | 2002 | no | noaa-tree-4008 | Meriläinen, J., Lindholm, M., Timonen, M. |
| 61.83 | 23.48 | *P. abies* | 1860 | 1978 | yes | noaa-tree-4478 | Schweingruber, F.H. |
| 61.85 | 28.9 | *P. sylvestris* | 1864 | 1993 | no | noaa-tree-3999 | Meriläinen, J., Lindholm, M., Timonen, M., Huttunen, P. |
| 61.85 | 28.9 | *P. sylvestris* | 1798 | 1993 | no | noaa-tree-3992 | Meriläinen, J., Lindholm, M., Timonen, M., Huttunen, P. |
| 61.87 | 28.82 | *P. abies* | 1815 | 2001 | no | noaa-tree-4003 | Meriläinen, J., Lindholm, M., Timonen, M. |
| 61.92 | 26.02 | *P. sylvestris* | 1750 | 2000 | no | noaa-tree-3973 | Melvin, T.M. |
| 61.92 | 29 | *P. sylvestris* | 1851 | 2002 | no | noaa-tree-3989 | Meriläinen, J., Lindholm, M., Timonen, M., Kolström, T. |
| 61.93 | 25.72 | *P. sylvestris* | 1750 | 2000 | no | noaa-tree-3976 | Melvin, T.M. |
| 61.93 | 28.98 | *P. sylvestris* | 1865 | 2001 | no | noaa-tree-3987 | Meriläinen, J., Lindholm, M., Timonen, M., Kolström, T. |
| 62.58 | 31.17 | *P. sylvestris* | 1779 | 1978 | yes | noaa-tree-4434 | Schweingruber, F.H. |
| 62.85 | 25.48 | *P. sylvestris* | 1643 | 1978 | yes | noaa-tree-4606 | Schweingruber, F.H. |
| 62.85 | 25.48 | *P. sylvestris* | 1602 | 1983 | no | noaa-tree-2845 | Briffa, K. |
| 62.93 | 31.38 | *P. sylvestris* | 1809 | 1983 | no | noaa-tree-3200 | Eronen, M. |
| 62.98 | 31.3 | *P. sylvestris* | 1589 | 1983 | no | noaa-tree-3201 | Eronen, M. |
| 62.98 | 31.3 | *P. sylvestris* | 1831 | 1983 | no | noaa-tree-3199 | Eronen, M. |
| 63.02 | 31.43 | *P. sylvestris* | 1784 | 1983 | no | noaa-tree-3202 | Eronen, M. |
| 63.1 | 25.48 | *P. abies* | 1818 | 1978 | yes | noaa-tree-4472 | Schweingruber, F.H. |
| 63.1 | 29.63 | *P. sylvestris* | 1882 | 2001 | no | noaa-tree-3998 | Meriläinen, J., Lindholm, M., Timonen, M., Kolström, T. |
| 63.1 | 30.63 | *P. sylvestris* | 1683 | 1984 | no | noaa-tree-3207 | Eronen, M. |
| 63.28 | 28.93 | *P. sylvestris* | 1821 | 2001 | no | noaa-tree-3993 | Meriläinen, J., Lindholm, M., Timonen, M., Kolström, T. |
| 63.68 | 29.88 | *P. sylvestris* | 1588 | 1983 | no | noaa-tree-3206 | Eronen, M. |
| 64.55 | 26.52 | *P. sylvestris* | 1839 | 1978 | yes | noaa-tree-4619 | Schweingruber, F.H. |
| 65.62 | 27.6 | *P. abies* | 1755 | 1978 | yes | noaa-tree-4441 | Schweingruber, F.H. |
| 66.32 | 25.15 | *P. sylvestris* | 1578 | 1983 | no | noaa-tree-2843 | Briffa, K. |
| 66.37 | 29.3 | *P. sylvestris* | 1758 | 2001 | no | noaa-tree-4005 | Meriläinen, J., Lindholm, M., Timonen, M., Eronen, M. |
| 66.37 | 29.43 | *P. abies* | 1726 | 1978 | yes | noaa-tree-4578 | Schweingruber, F.H. |
| 67 | 27.12 | *P. sylvestris* | 1670 | 1978 | yes | noaa-tree-4492 | Schweingruber, F.H. |
| 67 | 27.12 | *P. abies* | 1701 | 1978 | yes | noaa-tree-4491 | Schweingruber, F.H. |
| 67 | 27.25 | *P. sylvestris* | 1655 | 1983 | no | noaa-tree-2846 | Briffa, K. |
| 67.08 | 27.02 | *P. sylvestris* | 1550 | 2000 | no | noaa-tree-3975 | Melvin, T.M. |
| 67.22 | 26.82 | *P. sylvestris* | 1550 | 2000 | no | noaa-tree-3974 | Melvin, T.M. |
| 68 | 24.2 | *P. sylvestris* | 1657 | 1983 | no | noaa-tree-2841 | Briffa, K. |
| 68.03 | 24.1 | *P. abies* | 1782 | 1978 | yes | noaa-tree-4580 | Schweingruber, F.H. |
| 68.07 | 27.2 | *P. sylvestris* | 1740 | 2001 | no | noaa-tree-4021 | Meriläinen, J., Lindholm, M., Timonen, M. |
| 68.13 | 27.45 | *P. sylvestris* | 1560 | 1983 | no | noaa-tree-2855 | Briffa, K. |
| 68.45 | 28.07 | *P. sylvestris* | 1562 | 1983 | no | noaa-tree-2848 | Briffa, K. |
| 68.53 | 28 | *P. sylvestris* | 1659 | 1992 | no | noaa-tree-4014 | Meriläinen, J., Lindholm, M., Timonen, M., Eronen, M. |
| 68.67 | 25.87 | *P. sylvestris* | 1536 | 1983 | no | noaa-tree-2834 | Briffa, K. |
| 68.83 | 27.25 | *P. sylvestris* | 1410 | 2001 | no | noaa-tree-3991 | Meriläinen, J., Lindholm, M., Timonen, M., Eronen, M. |
| 68.87 | 26.88 | *P. sylvestris* | 1560 | 1983 | no | noaa-tree-2827 | Briffa, K. |
| 68.92 | 28.48 | *P. sylvestris* | 1622 | 2001 | no | noaa-tree-3994 | Meriläinen, J., Lindholm, M., Timonen, M., Eronen, M. |
| 69.32 | 28.13 | *P. sylvestris* | 1532 | 1983 | no | noaa-tree-2857 | Briffa, K. |
