# Supplementary figures and images for: Reliability of temperature signal in various climate indicators from northern Europe
Source: PLoS One. 2017 Jun 29;12(6):e0180042. doi: 10.1371/journal.pone.0180042 (PMC5491121; doi:10.1371/journal.pone.0180042)

# Jyväskylä

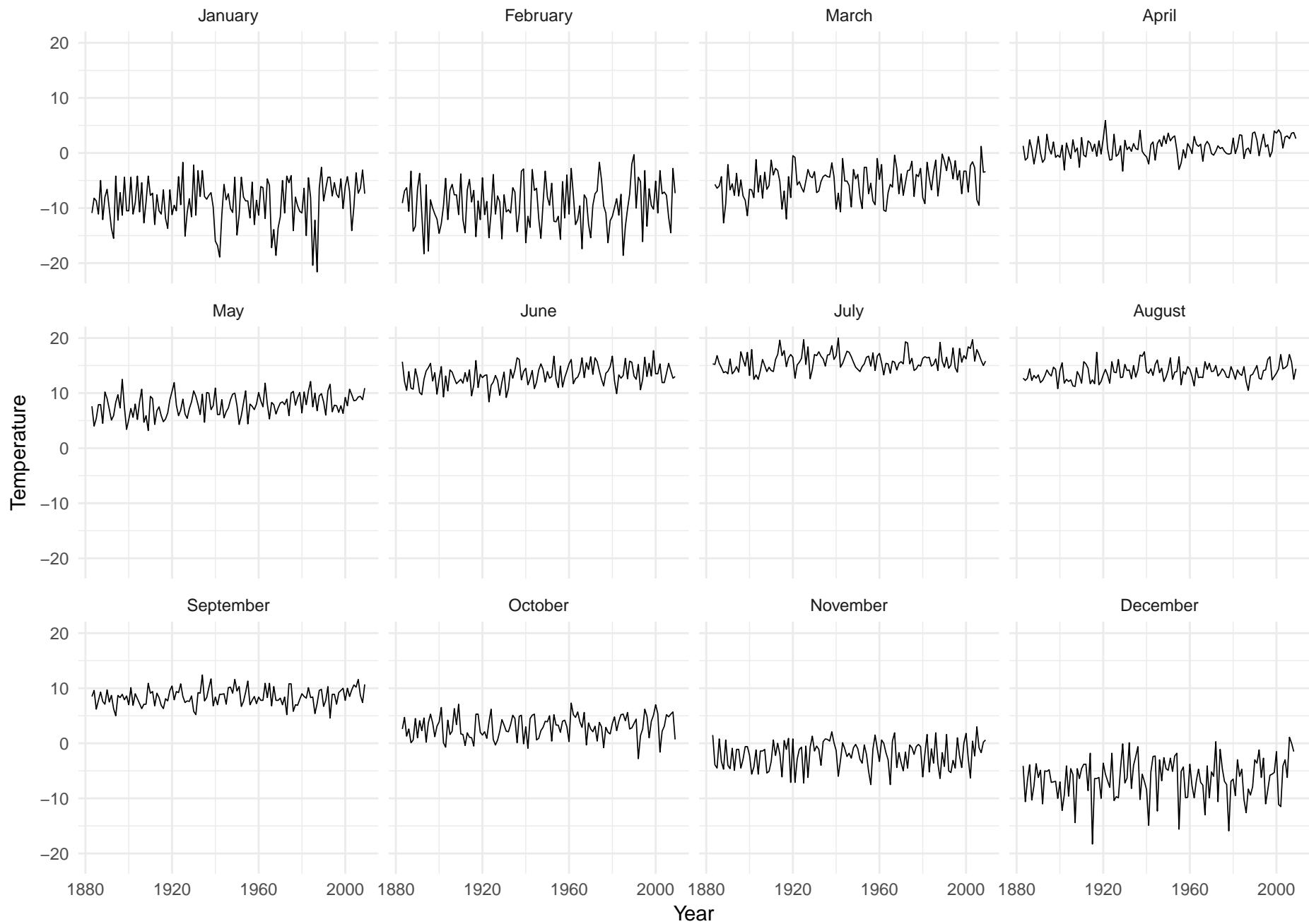

Supplement: S1 Fig — (PDF) [file pone.0180042.s002.pdf]

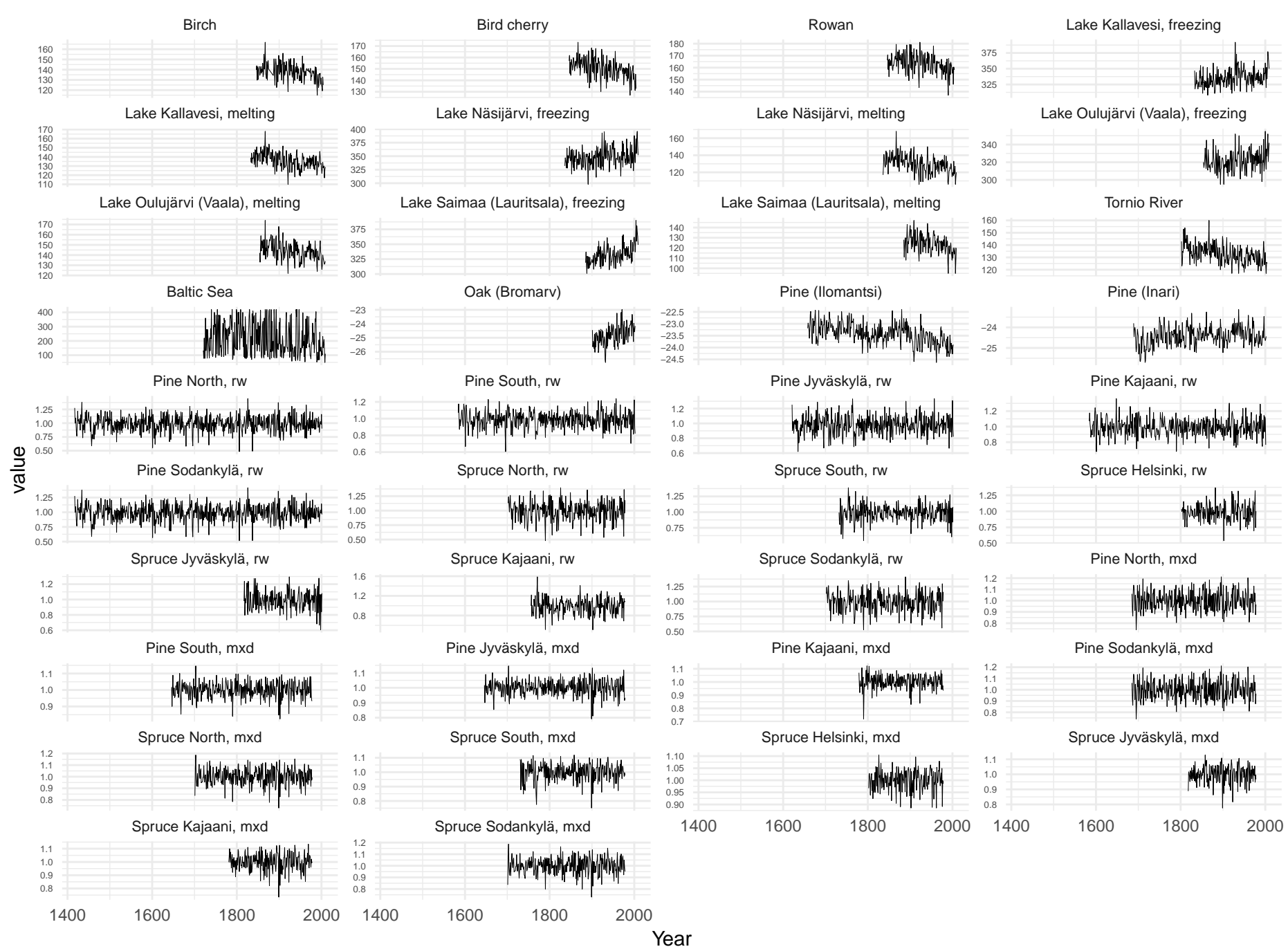

Supplement: S2 Fig — (PDF) [file pone.0180042.s003.pdf]
